# Accuracy of Complete-Arch Scans Obtained by Intraoral Scanner and Smartphone Three-Dimensional Scanning Applications With Different Smartphone Position Setups: An In Vitro Study

**DOI:** 10.7759/cureus.63471

**Published:** 2024-06-29

**Authors:** Yuhao Jiang, Hu Long, Suet Yeo Soo, Hetal Mavani, In Meei Tew

**Affiliations:** 1 Department of Restorative Dentistry, The National University of Malaysia, Kuala Lumpur, MYS; 2 Department of Orthodontics, West China Hospital of Stomatology, Sichuan University, Chengdu, CHN

**Keywords:** dental models, scan accuracy, mobile phone application, intraoral scan, digital dentistry

## Abstract

Introduction: The high cost of intraoral scanners (IOS) for complete-arch scans makes them less accessible for many dental practitioners. As a viable alternative, smartphone scanner applications (SMP) provide comparable scanning capabilities at a significantly low cost. However, there is limited data on the accuracy of SMP, especially when used in various smartphone positions. This study aimed to compare the three-dimensional (3D) and linear accuracy of complete-arch scans acquired by an IOS and SMP (KIRI Engine, KIRI Innovations, Guangdong, China) at three shooting angles (0°, 45°, and 90° for SMP_3A) and two shooting angles (30° and 60° for SMP_2A).

Methods: A stone dental cast was scanned with a laboratory scanner as a reference, with 11 scans performed by an IOS, SMP_2A, and SMP_3A. In 3D analysis, trueness and precision were evaluated through superimposition with the reference scan and within each group, respectively, using the best-fit algorithm of Geomagic Wrap software (3D Systems, Inc., Rock Hill, SC). Trueness in linear discrepancy was assessed by comparing the occlusal-cervical and mesiodistal dimensions of reference teeth (canine, premolar, and molar), intercanine width, and intermolar width on the digital casts to measurements of the stone cast, while precision was measured using the coefficient of variance. Differences between groups were analyzed using the Friedman test, followed by the Dunn-Bonferroni post hoc test with a significance level set at 0.05.

Results: IOS exhibited significantly lower trueness than SMP_2A (p = 0.003) with significantly greater width discrepancies on canines (p = 0.001) and molars (p < 0.001). Discrepancy patterns differed among the three scanning methods. The IOS showed greater discrepancies on the occlusal surfaces of posterior teeth. While SMP_3A demonstrated higher variation on the palatal surfaces and interproximal areas of posterior teeth. For precision, SMP_3A (p = 0.028) and SMP_2A (p = 0.003) showed a significantly lower precision in 3D analysis, but a comparable reproducibility in linear measurement to IOS.

Conclusion: TRIOS IOS (3Shape, Copenhagen, Denmark) exhibited lower trueness in 3D and linear accuracy analyses for complete-arch scans. The positions of the smartphone significantly enhanced trueness at the undercut region. SMP_2A and SMP_3A can be a potential alternative for precise linear measurement in complete-arch scans with selective use.

## Introduction

Digital technology has brought about a significant transformation in our dental practice by streamlining the conventional impression workflows. The use of digital impressions addresses the challenges associated with the storage of gypsum dental casts [1], dental cast damage [2], and difficulties in communication among dental professionals, patients, and dental technicians [3]. Furthermore, the remarkable accuracy of dental casts obtained through digital technologies is essential to achieve well-fitting and durable dental prostheses [4,5].

The term “accuracy” is defined by the International Organization for Standardization standard 5725-2, consisting of both trueness and precision. Trueness refers to the closeness of agreement between the arithmetic mean of a large number of test results and the true value. Precision refers to the closeness of agreement between test results [6].

Three-dimensional (3D) dental casts can be obtained using either direct or indirect techniques. Intraoral scanners (IOS) are commonly used for directly capturing both hard and soft tissues within the oral cavity. These individual images of oral tissues are subsequently stitched together via specialized software, resulting in chair-side 3D dental casts. The accuracy of intraoral scans varies significantly, influenced by several factors, including the scanning technology [7], the chosen scanning strategy [3,8], lighting conditions [9], the clinician’s experience [10], and scanned area distance [11,12]. On the other hand, 3D dental casts can be indirectly generated through optical-based desktop scanner systems, which have demonstrated superior accuracy compared to direct intraoral scanning [12,13]. Therefore, 3D dental casts acquired via desktop scanners are often used as reference casts in studies assessing the accuracy of 3D dental casts [14,15]. However, the high cost of dental scanners is the main limitation, making them inaccessible to many dental clinicians.

With the continual advancement in digital photography, low-cost 3D smartphone scanner applications (SMPs) have gained attention in dentistry, particularly for facial scanning. However, the accuracy of these applications has been inconsistent. Thurzo et al. suggested that facial scanning using SMP has limited clinical applicability due to higher 3D trueness deviation, exceeding 3 mm in most virtual facial regions compared to referenced computed tomography scans [16]. A similar outcome was reported by Elbashti et al. [17]. Conversely, Rudy et al. demonstrated that SMP outperformed portable 3D cameras, exhibiting higher trueness and precision within 0.5 mm [18].

SMP works in a manner similar to conventional photogrammetry techniques, capturing a series of overlapped photos with a smartphone camera. These photos are then processed in cloud-based photogrammetry software to create 3D objects. Photogrammetry technology has been reported as a reliable alternative to IOS for acquiring implant scans [19]. However, there is a lack of studies on the accuracy of SMP with photogrammetry technology for complete-arch scans as compared to IOS, particularly regarding the influence of smartphone positioning with different shooting angles, which significantly influences scan depth, details, and accuracy.

Therefore, this in vitro study aims to compare the accuracy, including both 3D and linear discrepancies, of complete-arch scans obtained by IOS and SMP using two different pre-determined smartphone position setups with three shooting angles (0°, 45°, and 90°) and two shooting angles (30° and 60°). The null hypothesis was that the SMP with two smartphone position setups would provide equivalent trueness and precision to IOS in complete-arch scans.

## Materials and methods

Reference scan obtained via a laboratory scanner

This in vitro study was carried out at the Prosthodontic Laboratory, Faculty of Dentistry, the National University of Malaysia. A sample size of 11 per group was determined using an effect size of 1.00 [20], a power of 90%, and a significance level set at 0.05. A completely dentate stone cast was digitized using a laboratory scanner (3Shape E3, 3Shape, Copenhagen, Denmark) with an accuracy of 7, and a stereolithography (STL) file was created and served as a reference file.

Three-dimensional dental casts obtained by IOS

In the IOS group, the stone cast was scanned using an IOS (TRIOS 3, software version 19.2.2, 3Shape, Copenhagen, Denmark) following the manufacturer’s instructions. The scanning started on the occlusal surface of the left second molar, sweeping along the occlusal plane. During the scan, careful manipulation was taken to gently wiggle the scanner while advancing toward the central and proceeding to the occlusal surface of the contralateral right second molar. The scanner was then slowly turned buccal to the right second molar to complete the buccal swipe. Then the scanner was rolled to the palatal side for a thorough swipe. To include the palate in the scan, the scanner was positioned behind the incisors, executing a side-to-side swipe in the distal direction. The scanning process was repeated 11 times, resulting in a total of 11 STL files. The time taken for these 11 digital scans was recorded by an operator (Y.H.J.) in minutes. All digital scans were performed by an operator with three years of clinical experience with intraoral scanning.

Three-dimensional dental models obtained by SMP_3A and SMP_2A

**Figure 1:**
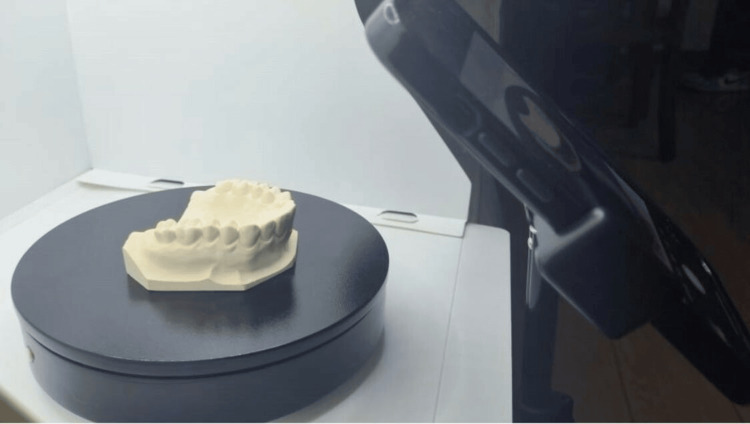
The smartphone was mounted on a tripod and positioned 15 cm away from the stone dental cast.

**Figure 2:**
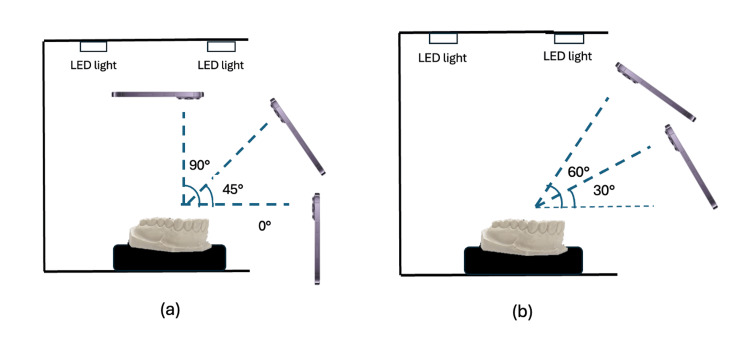
(a) The smartphone camera position setup in SMP_3A using three shooting angles 0°, 45°, and 90°. (b) The smartphone camera position setup in SMP_2A using two shooting angles 30° and 60°. SMP: smartphone scanner application; LED: light-emitting diode.

In the SMP group, the stone dental cast was positioned on a round tray above a motorized time-lapse rotating tripod within a light box equipped with light-emitting diode (LED) white lights (5500K, 5V). The smartphone camera (iPhone 14 Pro Max, Apple Inc., Cupertino, CA) with specifications including a 48-megapixel sensor, a resolution of 2556 x 1179 pixels, and a pixel density of 460 ppi was positioned on another tripod at a distance of 15 cm from the stone dental cast (Figure 1). Images of the stone dental cast were taken without a flashlight in two position setups. The first setup (SMP_3A) included three shooting angles: parallel to the occlusal plane, angled at 45° in relation to the occlusal plane, and perpendicular to the occlusal plane of the stone dental cast, as illustrated in Figure 2A. In the second setup (SMP_2A), two shooting angles set at 30° and 60° relative to the occlusal plane of the stone dental cast were used, as shown in Figure 2B. To ensure a substantial overlap of approximately 87% between consecutive photos, the stone dental cast was programmed to automatically rotate by 6° between each shot in a circular path. This approach resulted in a total of 180 images for SMP_3A and 120 images for SMP_2A, which were essential for reconstructing a 3D dental cast. The procedure was repeated 11 times and the time taken to capture these images was recorded by an operator (Y.H.J.) in minutes. Subsequently, all acquired images were imported to SMP (KIRI Engine, KIRI Innovations, Guangdong, China) to generate 11 3D dental casts for both SMP_3A and SMP_2A in STL format.

Accuracy of three-dimensional analysis

All STL files obtained from IOS, SMP_3A, and SMP_2A were assessed for trueness and precision using inspection software (Geomagic Wrap, v2021.2.2, 3D Systems, Inc., Rock Hill, SC). For trueness and precision assessment, two STL files were superimposed using a “best-fit algorithm,” and the 3D discrepancies were quantified using absolute average deviations calculated by the software. Trueness was assessed through a comparison of the reference file and test files, resulting in 11 absolute average deviations in each group. Precision was evaluated by comparing STL files within the test groups, yielding 55 absolute average deviations in each group. Colorimetric maps depicting the discrepancies of superimposed mesh between the reference scan and the test scans obtained from IOS, SMP_3A, and SMP_2A in occlusal and labial views were generated, with deviation values ranging from +3.00 mm to -3.00 mm, within a tolerance range of +0.02 mm/-0.02 mm (green).

Trueness and precision of linear dimensions

The measurements of horizontal (mesiodistal width, intercanine width, and intermolar width) and vertical dimensions (occlusal-cervical distance) were made of reference teeth (right canine, right first premolar, and right first molar) on stone cast using an electronic digital caliper. The horizontal and vertical dimensions of reference teeth on digital casts were measured with a built-in digital ruler in inspection software (Geomagic Wrap, v2021.2.2).

**Figure 3:**
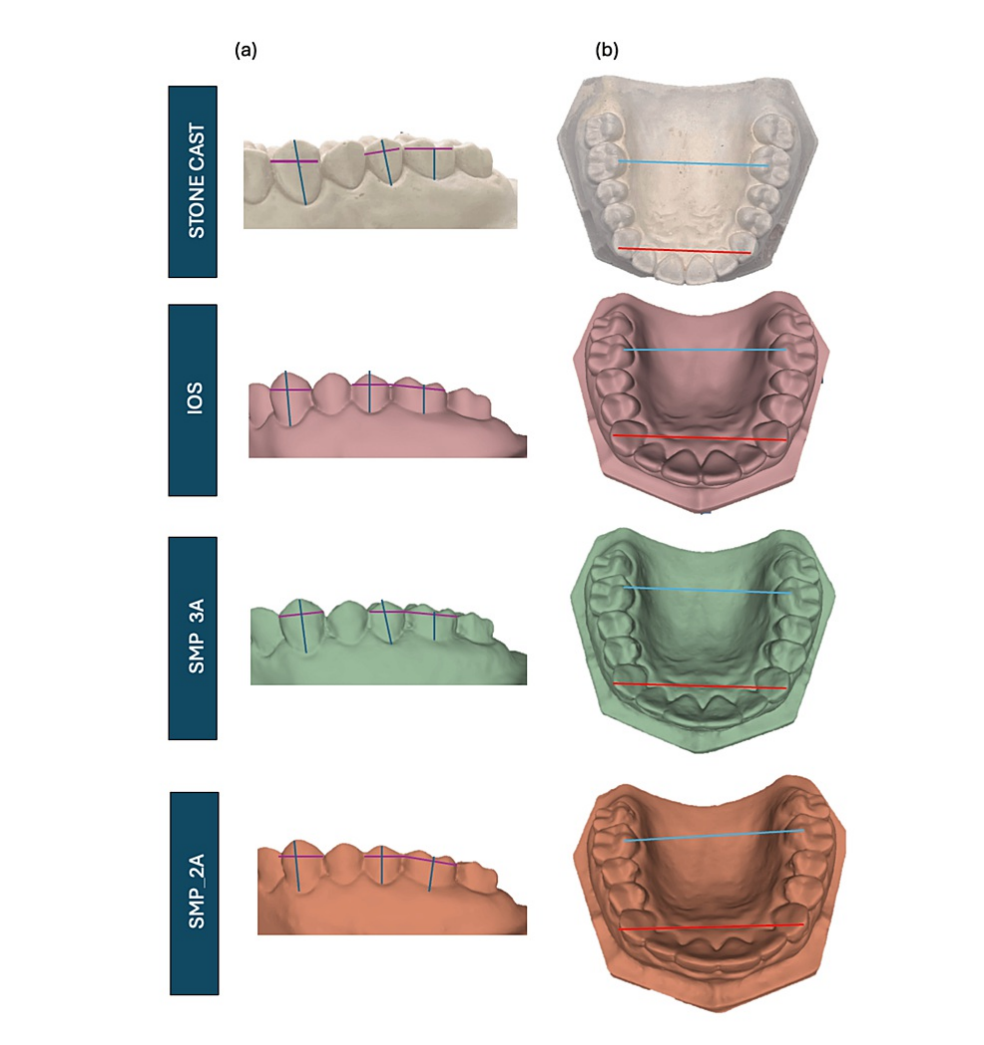
(a) Mesiodistal width (purple line) and occlusal cervical distance (dark blue line) of canine, premolar, and molar were measured in a stone cast, IOS, SMP_3A, and SMP_2A scans. (b) Intercanine width (red line) and intermolar width (light blue line) were measured in a stone cast, IOS, SMP_3A, and SMP_2A scans. IOS: intraoral scanner; SMP: smartphone scanner application.

The mesiodistal width was measured from the mesial to the distal contact point of reference teeth (Figure 3A). The occlusal-cervical distance was measured from the cusp tip to the gingival margin for canines and premolars, and from the buccal developmental groove to the gingival margin for molars (Figure 3A). The intercanine width was measured from the cusp tip of the right canine to the cusp tip of the left canine (red line in Figure 3B). The intermolar width was measured from the mesiolingual cusp tip of the right first molar to the mesiolingual cusp tip of the left first molar (blue line in Figure 3B).

All measurements were carried out by a trained investigator (Y.H.J.) and repeated three times. The investigator was blinded to scanning methods during the measurement. Trueness was determined by calculating the absolute mean deviations, obtained by subtracting the mean measurement on test digital casts from the mean measurement on the reference stone cast. The precision assessment involved calculating the coefficient of variation (CV) for each pair of linear measurements within the groups using the following formula:

CV = standard deviation/mean x 100%.

The intraclass correlation coefficient (ICC) was calculated for intra-observer reliability.

Statistical analysis

Statistical evaluation was performed using an analysis software program (IBM SPSS Statistics, v28, IBM Corp., Armonk, NY). The Shapiro-Wilk test confirmed that the data did not follow a normal distribution. Friedman test was used to assess the differences between groups in trueness and precision of the 3D and linear discrepancies. This was followed by the Dunn-Bonferroni test for post hoc analysis. The level of significance was set at a = 0.05.

## Results

Scanning time evaluation

A total of 11 3D dental casts were generated each by IOS, SMP_3A, and SMP_2A for trueness and precision comparison. The mean scanning time for IOS, SMP_3A, and SMP_2A was two minutes, nine minutes, and six minutes, respectively.

Trueness and precision comparison in three-dimensional analysis

**Figure 4:**
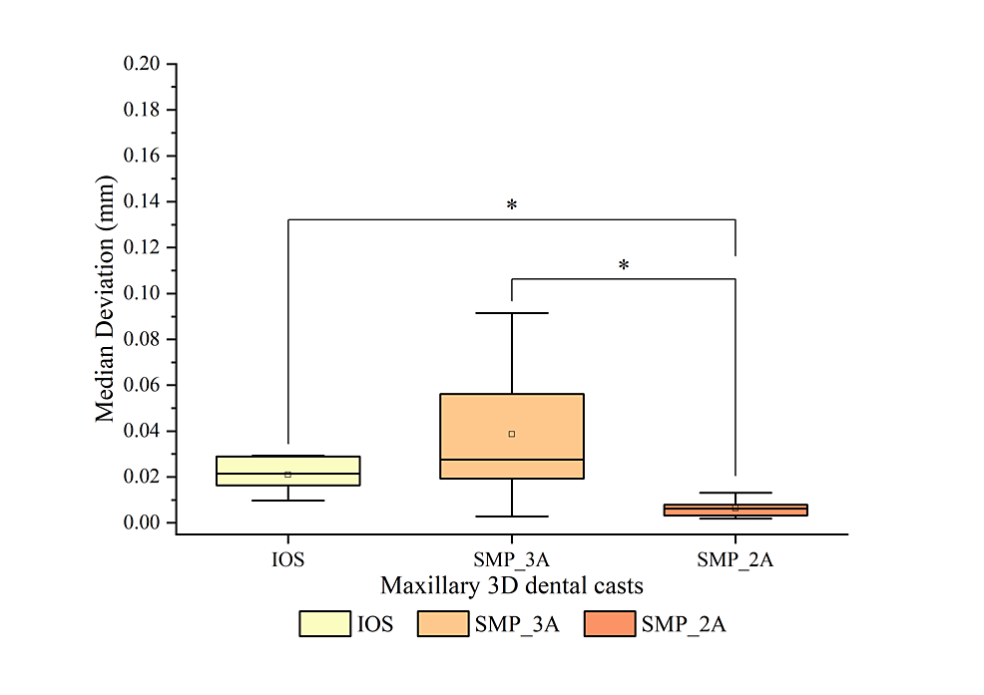
Comparing the 3D deviation in the trueness of 3D dental casts generated by IOS, SMP_3A, and SMP_2A. * Significant differences in the median deviation between the two groups (p < 0.05). IOS: intraoral scanner; SMP: smartphone scanner application.

The trueness in 3D comparison among the three test groups is displayed in Figure 4. It is noteworthy that SMP_2A showed significantly higher trueness (median: 0.006; IQR: 0.004) compared to IOS (median: 0.021; IQR: 0.013) (p = 0.003) and SMP_3A (median: 0.028; IQR: 0.541) (p = 0.001).

**Figure 5:**
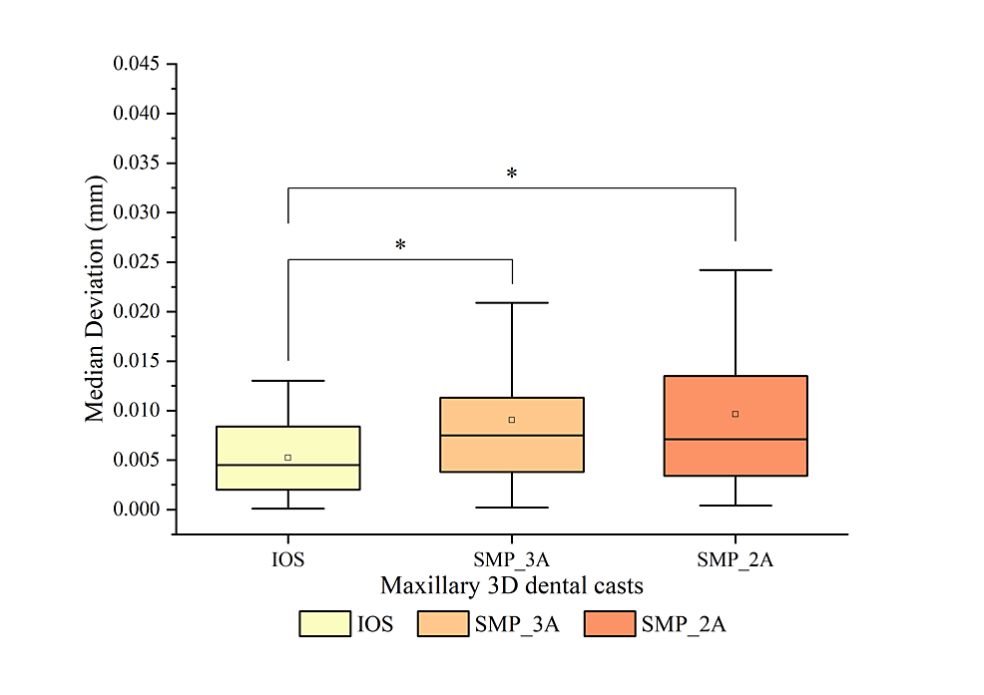
Comparing 3D deviation in the precision of 3D dental casts generated by IOS, SMP_3A, and SMP_2A. * Significant differences in the median deviation between the two groups (p < 0.05). IOS: intraoral scanner; SMP: smartphone scanner application.

The precision comparison among the three test groups is illustrated in Figure 5. It was observed that 3D dental casts generated by IOS had significantly higher precision (median: 0.005; IQR: 0.006) compared to SMP_2A (median: 0.007; IQR: 0.010) (p = 0.028) and SMP_3A (median: 0.008; IQR: 0.008) (p = 0.003).

Color map of mesh deviation between reference and test scans

**Figure 6:**
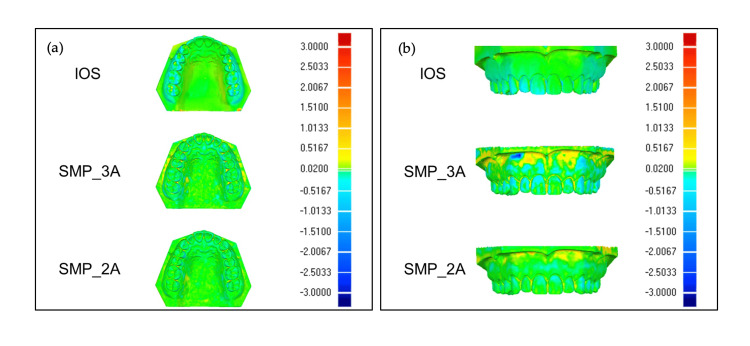
(a) Color map of mesh deviation between reference scan and test scans generated by IOS, SMP_3A, and SMP_2A in the occlusal view. (b) Color map of mesh deviation between reference scan and test scans generated by IOS, SMP_3A, and SMP_2A in the labial view. IOS: intraoral scanner; SMP: smartphone scanner application.

The color map of mesh deviation between reference scan and test scans generated by IOS, SMP_3A, and SMP_2A in the occlusal view is illustrated in Figure 6A. The IOS group showed an inward displacement (depicted in blue) on the occlusal surfaces of posterior teeth. Conversely, both SMPs exhibited variation on palatal surfaces (depicted by inward displacement in blue) and interproximal areas (depicted by outward displacement in yellow and red) of posterior teeth, with SMP_3A exhibiting greater deviation. The color map of mesh deviation between reference scan and test scans generated by IOS, SMP_3A, and SMP_2A in the labial view is displayed in Figure 6B. SMP_3A demonstrated greater discrepancies, with inward displacement (depicted in blue) at the cervical of anterior teeth and both inward (depicted in blue) and outward (depicted in yellow) displacement at the labial mucosal, compared to IOS and SMP_2A.

Accuracy of linear measurement

The intra-observer reliability was considered excellent with an ICC of 0.914. Median discrepancies in mesiodistal width of reference teeth, intercanine width, and intermolar width between the three test groups and reference stone cast are shown in Table 1. The median discrepancies of IOS were significantly higher when compared to SMP_3A and SMP_2A for the mesiodistal width of the canine (p = 0.001) and molar (p < 0.001). Median discrepancies in the occlusal-cervical dimension of reference teeth between the reference stone cast and the three test groups are shown in Table 2. There were no significant differences found in the height discrepancies among the three test groups.

**Table 1:** Comparison of median discrepancies of tooth and arch width between reference stone cast and three test groups. Different letters denote statistically significant differences between groups (p < 0.05). * Statistically significant (p < 0.05). IOS: intraoral scanner; SMP: smartphone scanner application; ICW: intercanine width; IMW: intermolar width.

	Median (IQR)			
	IOS (mm)	SMP_3A (mm)	SMP_2A (mm)	p-value
Canine	0.44 (0.40)^a,b^	0.12 (0.34)^a^	0.12 (0.20)^b^	0.001*
Premolar	0.27 (0.10)	0.13 (0.16)	0.13 (0.16)	0.050
Molar	0.41 (0.20)^a,b^	0.21 (0.20)^a^	0.21 (0.20)^b^	<0.001*
ICW	0.80 (0.60)	0.40 (0.70)	0.50 (0.30)	0.311
IMW	0.20 (0.50)	0.60 (0.40)	0.30 (0.40)	0.071

**Table 2:** Comparison of median discrepancies of occlusal-cervical dimension between reference stone cast and three test groups. Different letters denote statistically significant differences between groups (p < 0.05). * Statistically significant (p < 0.05). IOS: intraoral scanner; SMP: smartphone scanner application,

	Median (IQR)			
	IOS (mm)	SMP_3A (mm)	SMP_2A (mm)	p-value
Canine	0.06 (0.24)	0.24 (0.18)	0.12 (0.24)	0.234
Premolar	0.13 (0.08)	0.18 (0.35)	0.11 (0.17)	0.513
Molar	0.24 (0.20)	0.42 (0.10)	0.17 (0.30)	0.086

Repeatability coefficient of linear discrepancies

**Figure 7:**
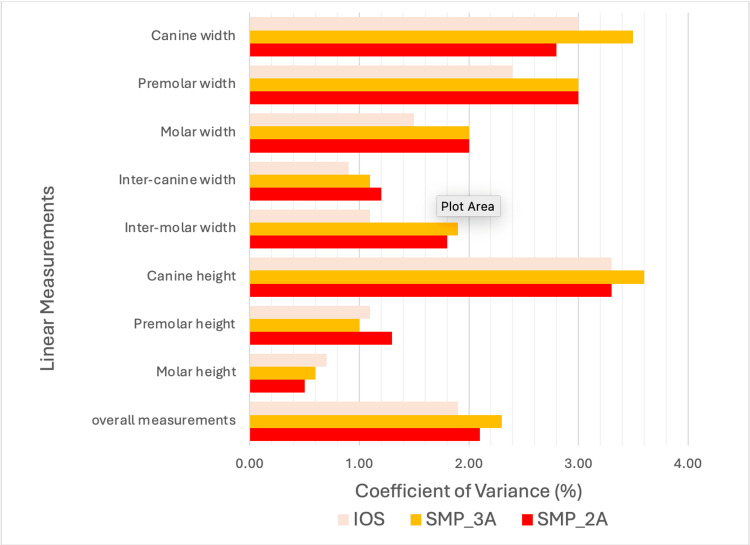
The percentage of coefficient of variance in linear discrepancies among IOS, SMP_3A, and SMP_2A. IOS: intraoral scanner; SMP: smartphone scanner application.

The coefficient of variance of linear discrepancies for IOS, SMP_3A, and SMP_2A is illustrated in Figure 7. All three test groups showed good repeatability with a coefficient variance of less than 4% in reference teeth. There were no significant differences found among the groups.

## Discussion

Smartphone-based photogrammetric technology has been well-established for digitizing facial surfaces [16-18] and auricular prostheses [20,21]. To the best of our knowledge, this study represents the first investigation into the feasibility of the low-cost smartphone 3D scanning application (KIRI Engine) for generating maxillary complete-arch scans. The study primarily focuses on KIRI Engine’s performance using two pre-defined smartphone position setups compared to IOS. Based on the results of the study, the null hypothesis, suggesting no significant difference among the three scanning methods, was partially rejected. Significant disparities were noted across the scanning methods in terms of the trueness of 3D and linear deviations. Additionally, significant variations were detected in the precision of 3D deviations.

In this present study, the reference file used for 3D analysis was obtained via a laboratory scanner, a widely accepted method known for its ability to accurately reproduce complex details and undercut areas in 3D casts, facilitating a better comparison with test groups [22]. The superimposition of two STL files was performed using the “best-fit algorithm,” which employs an iterative closest point (ICP) algorithm. This algorithm aligns the STL files without the influence of operator factors, thereby minimizing errors in the distance measurements between corresponding points on the files [23].

The findings of the study demonstrated that IOS exhibited a lower overall trueness in 3D comparison than SMP_2A. These findings align with other studies that have shown the suboptimal accuracy of IOS in obtaining complete-arch scans compared to conventional impression techniques [24] as well as photogrammetry scanning [22,25]. Previous studies have reported that larger scan distances using IOS could have greater deviations due to the stitching process of a series of 3D images, compared to quadrant scans [26-28]. The discrepancies were more noticeable in linear measurements, where IOS demonstrated significant width discrepancies, ranging from 0.41 mm to 0.44 mm on canines and molars as compared to both SMPs. This suggests that the accumulated scanning errors can contribute to horizontal scan errors, especially on the greater tooth surface areas in an arch [29]. Besides, scanning challenges were also noticed on the posterior occlusal surfaces, as illustrated in the color map in the present study. This is in line with a study by Pellitteri et al., who reported a similar distortion pattern in all three tested intraoral scanners [30]. Moreover, other potential factors negatively impacting the accuracy of IOS observed in the present study could be attributed to the operator's inadequate intraoral scanning experience and the lack of standardization in the scanning distance between the intraoral scanner tip and the recorded tooth surfaces, which aims to simulate the clinical situation.

On the other hand, photogrammetry-based scanning systems have the potential to overcome the limitations of IOS and improve the accuracy of complete-arch scans. Photogrammetry integrates all measured data from each image to generate a 3D cast using reference points, eliminating the need for image superimposition required in IOS, particularly during the “cut-off rescan” procedure to fill scan gaps, which can lower scan accuracy [31]. A few previous studies have reported favorable trueness results in 3D comparison using a photogrammetry system to digitize 3D cast [22,32]. Nevertheless, in the present study, only SMP_2A surpassed IOS in the trueness of 3D comparison. This suggests the significance of having optimal shooting angles and an appropriate smartphone camera setup to obtain detailed scans when using SMP for complete-arch scans.

Besides, the smartphone camera setup significantly impacts the overall trueness of 3D comparison in SMPs. The substantial discrepancies, which were particularly notable at the labial mucosa, cervical of anterior teeth, palatal surfaces, and interproximal of posterior teeth within SMP_3A, as shown in the color map, were attributed to underlying degrees of the undercut. These undercuts led to shadows during two-dimensional (2D) image acquisition, consequently distorting the 3D cast at this specific region [20]. The incorporation of two oblique shooting angles, set at 30° and 60° in SMP_2A, resulted in a remarkable reduction, nearly four-fold, of the discrepancies observed in SMP_3A, particularly at these undercut regions. This improvement is likely attributable to the selected two shooting angles in SMP_2A, facilitating access to these challenging undercut areas during photography, and thereby improving the accuracy of the scans.

In regard to precision, higher interquartile range values observed in both SMP_2A and SMP_3A indicated that overall precision in 3D comparison for both SMPs was significantly lower compared to IOS. This finding was inconsistent with studies using commercially available photogrammetry systems, which showed superior overall precision in 3D comparison than IOS [22,25]. The improved precision in commercially available photogrammetry systems can likely be attributed to the use of high-precision cameras, a standardized fixed acquisition position, and precise measurements through optical reference markers, which could be possible inherent limitations in SMP [33]. Nevertheless, both SMPs demonstrated comparable precision in linear measurements to IOS, with good reproducibility. This can be possibly due to the landmarks used for linear measurement in this study mainly located at the coronal third of the 3D casts, where scans exhibited minimal distortion. Zotti et al. who utilized open-source software to generate 3D casts from multiple 2D images also reported the effectiveness of a photogrammetric-based scanning system in achieving reproducible linear measurements [34]. Despite a different outcome between 3D and linear comparison due to inherent differences in measurement nature, these three scanning techniques could potentially offer precise scanning capabilities in selected applications involving maxillary complete-arch scans.

The scanning time was compared among the three scanning techniques in this study. It was observed that the TRIOS scanner using the confocal principle remained the fastest for obtaining complete dental arch scans when compared to time-consuming SMPs [35]. Notably, the scanning time for SMPs increases with the number of 2D images required for 3D reconstruction. However, some studies explored reducing scanning time using smartphone camera video recording during image acquisition [36-38]. Further studies are needed to compare the effectiveness of videogrammetry and IOS in generating 3D dental casts.

Our study has some limitations that should be acknowledged. Firstly, our in vitro study focused on assessing the accuracy of maxillary complete-arch scans based on a reference study cast using a specific SMP. To fully explore the capabilities of SMP, further studies should encompass diverse clinical scenarios, involving partially dentate and completely edentulous scans, with larger sample sizes and multiple operators for inter-operator variability assessment. Furthermore, in vivo studies are needed to support the clinical adoption of SMPs, addressing challenges in capturing detailed images of posterior teeth, essential for achieving superior 3D model reconstruction in clinical practice.

## Conclusions

Within the limitations of the study, it appears that IOS exhibits lower trueness in 3D analysis with greater linear deviations on larger tooth surface areas in complete-arch scans. The incorporation of two oblique shooting angles in SMP_2A offers advantages in improving trueness at areas with undercuts compared to SMP_3A. SMP_2A and SMP_3A exhibited good repeatability in linear measurement comparable to IOS, but lower precision in 3D analysis to IOS, indicating that all three tested groups can be potential precise scanning techniques for complete-arch scans with selective use.
